# Coupled crystal orientation-size effects on the strength of nano crystals

**DOI:** 10.1038/srep26254

**Published:** 2016-05-17

**Authors:** Rui Yuan, Irene J. Beyerlein, Caizhi Zhou

**Affiliations:** 1 Department of Materials Science and Engineering, Missouri University of Science and Technology, Rolla, MO 65409, USA; 2 Theoretical Division, Los Alamos National Laboratory, Los Alamos, NM 87545, USA

## Abstract

We study the combined effects of grain size and texture on the strength of nanocrystalline copper (Cu) and nickel (Ni) using a crystal-plasticity based mechanics model. Within the model, slip occurs in discrete slip events exclusively by individual dislocations emitted statistically from the grain boundaries. We show that a Hall-Petch relationship emerges in both initially texture and non-textured materials and our values are in agreement with experimental measurements from numerous studies. We find that the Hall-Petch slope increases with texture strength, indicating that preferred orientations intensify the enhancements in strength that accompany grain size reductions. These findings reveal that texture is too influential to be neglected when analyzing and engineering grain size effects for increasing nanomaterial strength.

It is well known that polycrystalline materials with smaller average grain sizes are stronger^1^. Most often, it is observed that the strength scaling with grain size D follows a Hall-Petch relationship


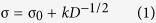


where σ_0_ is the friction resistance for dislocation movement within polycrystalline grains and *k* is the Hall-Petch slope. Yield strength and flow stress result from the motion of dislocations on specific crystallographic planes. Within the grains, dislocations nucleate, propagate, recover, and collect in pile-ups or as subgrain structures^2^. Many theories have been developed to link these dislocation processes to the observed Hall-Petch relation^1^,^3^,^4^,^5^. Most, but not all, suggest that the empirical, pervasive Hall-Petch law involves a grain size effect on the size of the dislocation pile-ups or the accumulation of dislocations within grains. The Hall-Petch value, *k*, in these cases is related to the shear stress required to push the leading dislocation to transmit through the grain boundary (GB).

In addition to grain size, strength also depends on the texture of the material, the distribution of crystallographic orientations^6^. The number of active slip systems and the distribution of shears among them depend on the orientation of the grains. The effect of texture on the strength scaling has received considerably less attention. Previously, its effect has been appreciated by introducing the Taylor factor *m*_T_ within the Hall-Petch slope *k*^1^, in order to relate the resolved shear stress needed to move a dislocation across the GB to a macroscopic uniaxial stress for the material. This factor is calculated as the inverse of an effective Schmid factor and hence varies with texture. As *m*_T_ increases, the Hall-Petch value will increase.

Over the past several years, numerous experiments have reported that the Hall-Petch scaling applies even to materials with grain sizes that lie in the nano-scale regime (~10 nm < *D *< 100 nm)^7^,^8^,^9^,^10^,^11^,^12^,^13^,^14^,^15^,^16^,^17^,^18^,^19^,^20^,^21^,^22^. The reason it occurs for both coarse- and nano-grained polycrystalline materials is not intuitive. *In-situ* transmission electron microscopy (TEM) and molecular dynamics (MD) studies of deformed nanocrystalline materials have provided insight into how dislocations move within the grains and they suggest that the predominant kinetics is very different than that in coarse-grained materials. First, they have revealed that dislocation-dislocation interactions and dislocation accumulations seldom happen within nano-sized grains^23^. Second, the plastic deformation of nanocrystalline (NC) metals instead proceeds by discrete and separated dislocation slip events, in which a dislocation originates at a grain boundary, glides across the grain unhindered, and recovers at the opposing grain boundary^3^,^23^,^24^. None of these phenomena predominate in deformed coarse-grained materials, yet Hall-Petch scaling is seen to prevail in most NC and coarse grained (CG) material studies. It may arise simply because over the nano-grain size range, NC material yield strength is nevertheless related to dislocation processes operating within the grains. Armstrong^25^ suggested that the Hall-Petch model occurs in NC materials since grain boundaries still resist dislocation motion. When grain sizes decrease to nano-scale, the number of pile-up dislocations decreases, transitioning from a multiple dislocation pile-up to a single dislocation loop expanding against the grain boundary.

When crystallographic slip occurs, texture should matter when material strength is a concern. In contrast to CG materials, texture effects on NC material strength have not been a focus and attention is usually given to improving processing techniques by reducing the grain size, chemistry, and grain boundaries. However, these different NC material processing methods inevitably induce noticeably diverse crystallographic textures^26^. A few recent studies, dedicated to analyzing both texture and size scaling in NC materials, have reported a strong effect of texture. In nanotwinned (NT) columnar Cu, the yield stresses measured when testing parallel and perpendicular to the twin boundaries differed by 30%^27^. The yield stress in NT Ag films showed a strong dependence on the intensity of the epitaxial {111} texture component^28^. Dalla Torre *et al*.^7^ investigated two commercial NC Ni samples with the same grain size (~20 nm) and clearly demonstrated that the strong (100) and weak (100) initial textures can result in non-negligible differences in yield strength, ultimate tensile strength and plastic strain. Godon *et al*.^14^ studied the effect of crystallographic texture on the relationship between grain size and flow stress in NC Ni. Their experiment results showed three distinct regimes in the Hall-Petch plot, corresponding to samples with a (100) texture, (110) texture and random texture. They concluded that differences in Hall-Petch slope resulted from differences in the deformation mechanisms induced by texture. In most of the above cases, samples with different grain sizes had different textures. Understanding the degree of coupling between texture and nanograin deformation is important for interpreting measurements of strength-grain size scalings in nanocrystalline materials.

The strong texture effects seen in these studies are signatures of crystallographic slip mediated deformation. It is natural then to postulate that texture would influence the strength scaling observed in NC materials. Texture effects could be even stronger in NC metals than they are in coarse-grained (CG) metals due to the lack of strengthening from dislocation storage within the grains. Addressing this hypothesis computationally encounters a length-scale dilemma. On the one hand, spatially resolved crystal plasticity techniques like crystal plasticity finite element (CPFE) have been developed to calculate the effects of texture and texture evolution on the strength of polycrystals^29^. However, nearly all crystal plasticity based models assume that slip initiates and propagates homogeneously within the grains, without resolving the individual contributions of the dislocations^30^. While appropriate for CG materials, this assumption is not adequate for NC materials when the size of the dislocation loop becomes comparable to that of the grain. On the other hand, there are many atomic-scale or dislocation based models that account for the motion of single dislocations or a collection of distinct dislocations within nanocrystalline materials^31^. However the time and length scales of these techniques are too short for accessing the interplay of texture and slip in NC materials during laboratory mechanical testing.

Recently, we developed a CPFE model for NC materials, in which glide of a single dislocation is modeled as a discrete slip event, which initiates via its emission from a grain boundary and ends with its absorption at the opposing grain boundary. Glide on multiple planes is permitted and dictated by the stress state in the grain and its current orientation. For NC Ni, it was shown that the discrete-CPFE predicted a Hall-Petch scaling in spite of no pile ups or dislocation storage within the grains and the fact that the activation stresses for individual dislocations from GBs scaled as ln (*D*)/*D* and not *D*^−1/2 ^32.

In this work, the discrete-CPFE model is used to understand the effect of texture on the strength scaling with grain size. Calculations are performed for NC Cu and Ni with grain sizes ranging from 10 nm to 100 nm, sizes for which dislocation storage within the grains is minimal, and for different starting textures, from random to some commonly observed textures formed by deposition or deformation processing. We will show that the model predicts emergence of a *D*^−1/2^ Hall-Petch scaling in yield strength for all initial textures and materials studied. For stronger initial textures, the Hall-Petch slope increases, indicating greater strength sensitivity to grain size reduction. An important consequence of this result is that the plastic anistoropy associated with a textured material increases as the grain size reduces. These findings indicate that is important to consider texture when analyzing size effects in NC material strength and can further help in the design of stronger and more formable NC materials.

## Discrete-CPFE

Crystal plasticity theory based modeling is needed if we wish to study texture effects. In this class of models, two general approaches exist: mean-field and full-field techniques, such as crystal plasticity finite element (CPFE)^33^. The latter, although the more computationally intensive, calculates spatially resolved mechanical fields as a result of grain-grain interactions and differences in orientation across grain boundaries. In both approaches, the grains are assumed to deform by homogeneous slip, where dislocations move as statistical distributions independent of their proximity to the grain boundaries. The significant deviation made in the present CPFE model is that slip occurs in discrete slip events, where individual dislocations are emitted from a GB and absorbed at an opposing one. The intent is to best capture the discrete nature of dislocation motion in NC metals, where grain sizes and dislocation lengths become comparable.

Within the current CPFE framework, a rate-dependent elasto-viscoplastic constitutive model is used^34^. The elastic contribution accounts for cubic anisotropy. For the visco-plastic contribution, the following power-law flow rule is adopted to relate the shear strain rate produced by an active slip system to the shear stress resolved on that system:


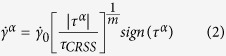


where 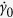 is a reference shear strain rate, 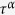 is the RSS on slip system 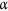, 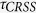 is a characteristic activation stress, and *m* is the strain rate sensitivity exponent. Most face centered cubic (FCC) metals deform using only the twelve {111} <110> slip systems^2^. In our calculations, all systems are made available in each grain and activation of multiple slip systems simultaneously is permitted.

**Figure 1:**
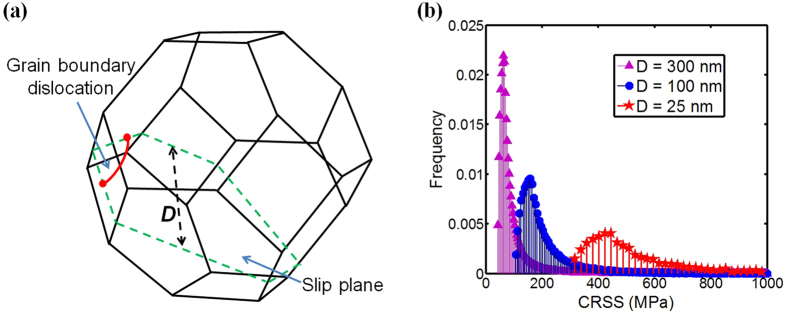
(**a**) Schematic of a dislocation source emanating from a grain boundary triple junction in a nanograin. The grain is embedded in a polycrystal and the grain boundary facets that it makes with the neighboring grains give it a shape of a tetrakaidecahedron; (**b**) Comparison of the probability density distribution of CRSS in NC Cu with grains sizes of 25 nm, 100 nm and 300 nm.

Within the model, the shape of the nanograins is assumed as a tetrakaidecahedron^35^, that is, a truncated octahedron as shown in Fig. 1(a). Most of the slip planes consequently take on a hexagon cross-section (while others may have a pentagon cross-section). Grain boundary sources take the form of a double-pinned dislocation segment of length *L* emanating from one of the six triple junctions of the plane. This grain boundary defect begins as a small embryo of activation length *L*_0_ on the order of the Burgers vector^32^. The embryonic dislocation expands until it is pinned at two ends by obstacles lying within the grain boundary^32^.

Growing the segment, now of length *L* (Fig. 1(a)) further requires a stress to bow out the dislocation between its two pinning points into the crystal to an unstable configuration, beyond which it can propagate into across the crystal without further increases in stress. Given the length of the double-pinned segment *L*, the characteristic stress to bow out the segment to an unstable configuration is given by Foreman’s formula^36^:


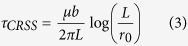


where *μ* is the effective shear moduli of the crystal and *r*_0_ is the core radius of the dislocation.

The value of *L* varies from *L*_0_ to *D*, the grain size. Assuming that any point within the grain boundary can be a pinning point leads to a statistical distribution in *L* and hence 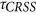
32. Because the upper limit of *L* equals *D*, the mean and variation of the 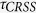 distribution depends on *D*. To demonstrate, Fig. 1(b) shows the statistical distributions for 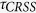 for *D* = 25 nm, 100 nm and 300 nm and using values associated with Cu: *r*_0_ = 5b, *b* = 0.256 nm and *μ* = 48.3 GPa. As *D* decreases, the distribution shifts toward larger values and the dispersion increases. Thus, by virtue of statistical source lengths from grain boundaries, dislocation activation becomes harder (the statistical mean increases) and more variable (the statistical dispersion increases) as *D* decreases. In this model, *D* places a physical limit on the size of the dislocation sources and is shown to modify both the mean and variance of the 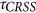 distribution.

Once emitted from a GB, the dislocation glides unhindered to the opposing grain boundary producing a shear strain given by^3^,^32^:


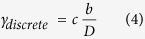


where *c* is a scaling parameter independent of grain size (*c* = 1.2 in this work), 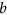 is the magnitude of the Burgers vector, and *D* is grain size. After crossing the grain, the dislocation is immediately absorbed (annihilated) at the boundary. Thus, dislocations are assumed not to pile up and produce a backstress.

After emission of a dislocation on slip system *α*, the original source has been exhausted and a new GB source must take its place in order to emit another dislocation on the same slip system. As the defect state of grain boundaries changes in time, the length of the new source and hence the 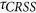 to emit a dislocation from it are not expected to be the same as that of the previous one. With the model, we capture this phenomenon by randomly assigning a new 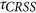 value from the corresponding 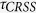 distribution for every new slip event. The time required for the discrete slip event to complete and hence a new 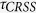 to be assigned can be calculated from equations (2) and (4).

It is worth highlighting that the discrete slip mechanism modeled here contains two ways in which *D* affects slip. First, dislocations are activated from sources of length *L*, where *L* can be no longer than *D* the diameter of a grain. Second, the slip distance can extend no further than *D* and the shear strain produced by each event depends on *D*.

## Material and Nanostructure

We use the model to investigate two types of NC materials, Ni and Cu, and different initial textures, all commonly studied in the literature. Electro- or physical vapor- deposition provides a highly textured NC material, where one set of planes is aligned along one direction and the remaining axes are otherwise randomly oriented in the other two orthogonal directions. We create a set of deposited {hkl} textures, where the {hkl} planes are aligned along the *z*-direction and nearly isotropic in the *x-y* plane. These include deposited {001}, {110}, and {111} textures. Severe plastic deformation (SPD) of two-phase composites is another way of forming nanomaterials^37^. These tend to produce textures where the interface planes correspond to {112} planes and the two in-plane directions of the NC Cu phase, the <111> and <110> directions, are highly aligned^38^. To model this initial texture, we build an SPD {112} texture in which these planes are aligned within a deviation of 20°. Last, other methods, such as ball-milling and consolidation processes^18^, produce nearly randomly oriented textures, and to understand these cases, as well as provide a natural reference situation, we numerically generate samples with a uniformly random texture.

Within the model, the kinetic equation (2), which introduces two parameters, the reference strain rate 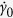 and rate sensitivity *m*, requiring characterization. For Ni, they are 10^−2^ s^−1^ and 0.1, respectively, which are taken from our prior work^32^. For Cu, we elect to use experimental stress-strain curves from NC Cu reported by Khan *et al*.^18^. There, the authors took great care in fabricating model-like samples that were sufficiently large for reliable mechanical testing and had a uniform grain size, making the data well suited for characterizing a model. Samples differing predominantly in their average grain size from 27 nm to 118 nm were tested. These experimental tests were conducted at a strain rate of 10^−4^ s^−1^ and therefore in the model, we set 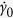 = 10^−4^ s^−1^.

**Figure 2:**
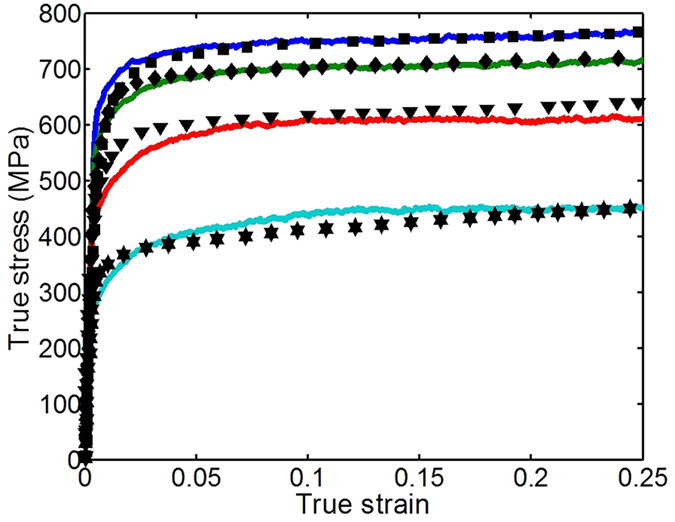
Comparison of experimental and calculated stress-strain curves for texture-free NC Cu with various grain sizes. (Experiment data are taken from ref. 18).

Consistent with these tests, the model of a randomly textured NC Cu is subjected to uniaxial compression at constant strain rate of 10^−4^ s^−1^. Figure 2 compares the calculated results using *m* = 0.1 for all grain sizes with the experiment results. With a single set of parameters, the model achieves good agreement in flow stress and hardening behavior for each test and most importantly, reproduces the pronounced grain size dependent strength in NC Cu. The value of *m* found here is well within the range of rate sensitivities reported for FCC metals^16^. In all calculations that follow, these parameters remain fixed.

## Results

**Figure 3:**
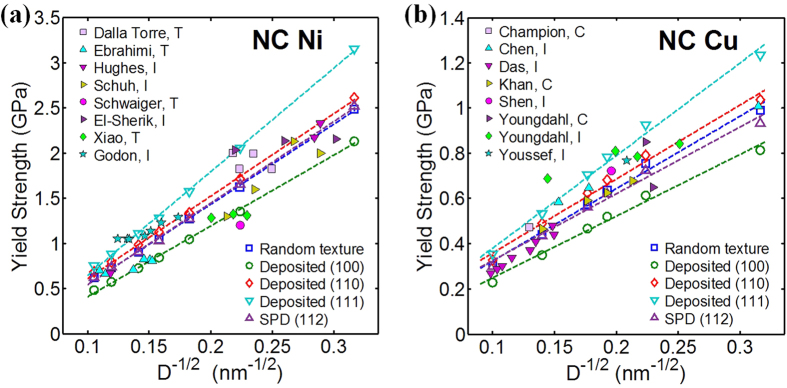
(**a**) Comparison of predicted yield strengths with experimental results on NC Ni from various groups; (**b**) Comparison of predicted yield strengths with experimental results on NC Cu from various groups. The dashed line represents the Hall-Petch fit to the calculated yield strength. Experimental data were obtained by nanoindentation marked with an “I”, compression marked with a “C” for tension marked with a “T”. (In nanoindentation tests, the yield strength was approximated as hardness divided by 3.0 41).

Figure 3(a) shows a Hall-Petch plot for the yield stress of NC Ni from different studies^7^,^8^,^9^,^10^,^11^,^12^,^13^,^14^. To first order, the combined experimental data show that as grain size decreases, strength increases. However, whether the Hall-Petch strength scaling emerges is not evident from the collection due to substantial dispersion among the measurements. Among samples of similar grain sizes, there may have been differences in texture, grain size dispersion, testing methods, and compositional purity and these factors can also affect yield stresses.

For comparison, Fig. 3(a) also presents the model results for the 0.2% yield stress taken from the calculated stress-strain curves for the different loading directions and initial textures. The calculation analyzes grain sizes no larger than 100 nm, since dislocation storage within these larger grains, not taken into account here, could contribute to further strengthening. Overall good agreement is achieved with the measurements in the sense that the different cases of starting textures and compression directions bound the data. Differences among the computed curves for different initial textures are substantial. Thus, the good comparison implies that initial texture differences in NC samples alone can result in a significant dispersion in strength.

As shown, the calculated yield stresses from each test follow a Hall-Petch scaling in grain size. The Hall-Petch slopes vary from 7.9 to 11.5 GPa ∙ nm^1/2^. These predicted Hall-Petch values match well with the range 5.0 to ~20.0 GPa ∙ nm^1/2^ commonly measured in NC Ni^39^,^40^.

**Figure 4:**
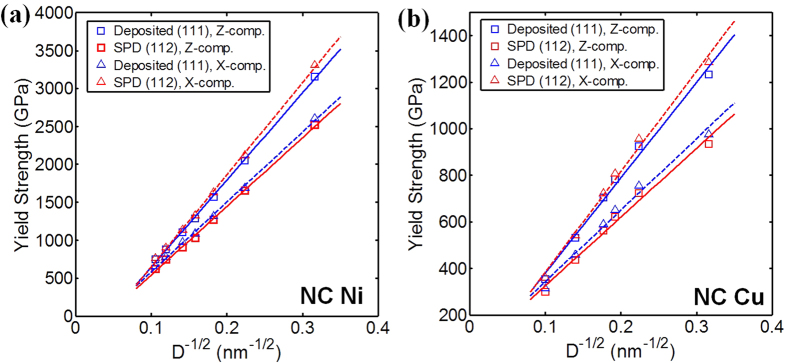
Comparison of predicted yield strengths under compression in z- and x-directions: (**a**) NC Ni (**b**) NC Cu.

In Fig. 4(a), we compare the scaling curves for the yield stress of NC Ni with the deposited (111) and SPD (112) textures under compression in the z- and x-directions, respectively. The results indicate pronounced yield anisotropy. The greatest strength occurs in cases in which the compression direction corresponds to the direction of (111) pole alignment (the z-direction in the deposited (111) texture and the x-direction in the SPD one). This direction dependence agrees with many single crystal experiments as well as dislocation dynamics and crystal plasticity simulations^42^,^43^,^44^. Compression in the x-direction of deposited (111) texture and z-direction of SPD (112) textures produces a lower strength. For both NC Ni and Cu, the strength in the x-direction of SPD (112) textures is closed to the strength in the z-direction of deposited (111) texture, since both of them carry the strong (111) texture.

The model indicates that texture and test direction not only affect yield stress and plastic anisotropy in yield, but also the Hall-Petch slope. The slope is higher for the loading direction-texture combinations giving the higher strengths. The finding implies that for a given sample, the direction leading to the highest strength would experience the highest gains in strength with reductions in grain size.

Figure 3(b) shows a Hall-Petch plot for the yield strengths of NC Cu collected from a large number of studies^15^,^16^,^17^,^18^,^19^,^20^,^21^. The experimental data plotted include tests from samples varying in textures and loading orientations. Over this range of grain sizes, smaller grain sizes lead to stronger material; however, considering all the data in combination, a pronounced strength scaling is not apparent. Neither a Hall-Petch nor an inverse Hall-Petch relationship can be concluded from the data.

In this plot, we also show the calculated yield strengths from five initially textured materials, with the random texture, deposited {111}, {100}, {110} textures, and SPD {112} texture. As seen in Ni, the differences in yield stress for Cu as a result of texture are significant, indicating a pronounced effect of texture. The comparison indicates that the model achieves good agreement with the data, and taken together, the model curves bound the data. Again, like Ni, the consistency between model and measurement imply that texture can contribute to the variation in the data observed among results reported in different studies.

One of the main results of this work is that a Hall-Petch scaling manifests in the computed yield strengths, for all initial textures and loading directions. The relationship is not a consequence of the grain size constraint on the length of dislocation pile-ups but on the length of dislocation sources in the grain boundaries. The source lengths and hence the stress to emit a dislocation are statistically distributed and fundamentally the critical stress does not follow a *D*^−1/2^ scaling. Rather the *D*^−1/2^ in macroscopic 0.2% yield and flow strength emerges from the collective response of the random emission of discrete slip events on multiple slip systems in an aggregate of grains.

**Figure 5:**
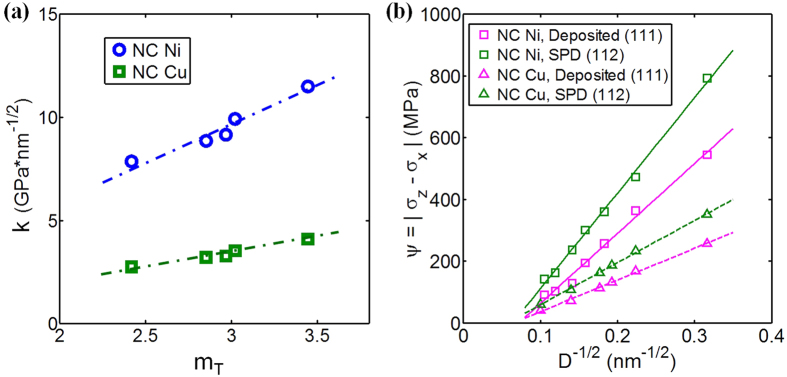
(**a**) Calculated Hall-Petch slope vs. *m*_T_ for all the cases studied; (**b**) comparison of the yield anisotropy, ψ = |σ_z_ − σ_x_| for deposited (111) and SPD (112) textures.

To quantitatively analyze the effects of texture on yield stress, it can be useful to calculate the Taylor factor *m*_T_. Here we define *m*_T_ as the inverse of the average of the five largest Schmid factors in each grain, which is then averaged over all grains. Accordingly, *m*_T_ is dependent solely on the relationship of the loading direction and texture. The values of *m*_T_ for different textures are listed in Table 1. Figure 5(a) is a plot of the calculated Hall-Petch slope with *m*_T_ for all the cases studied here. We observe in Fig. 5(a) a strong correlation between Hall-Petch slope, *k*, and *m*_T_, which is compelling because it suggests that the following Hall-Petch relationship can be applied to NC materials, via


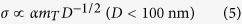


where *α* is a material dependent parameter, which is 3.59 GPa ∙ nm^1/2^ for Ni and 1.33 GPa ∙ nm^1/2^ for Cu. Thus the material parameter (*α*), grain size (*D*), and texture effects (*m*_T_) can be factored separately and embodied altogether into the Hall-Petch coefficient.

This scaling can explain why for a given texture, the yield anisotropy increases with reductions in *D*. If we express yield anisotropy as the difference in yield between the two test directions, say between z-direction and x-direction compression, *ψ* = |σ_z_ − σ_x_|, then from equation (5) we can expect that anisotropy would increase with decreasing grain size according to:


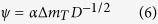


As *ψ* is proportional to 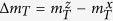 then a larger 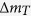 will induce a higher *ψ*. For a randomly texture NC material, 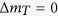 and hence the anisotropy is zero, as predicted in simulation. Consistent with this prediction, the yield anisotropy of the SPD (112) textured material is calculated to be stronger than that of the deposited (111) textured material as shown in Fig. 4(b).

## Discussion

The analysis shows that provided that the texture and loading direction with respect to the texture are fixed, a Hall-Petch scaling can arise. It also demonstrates that the variation in strength caused by different starting textures can be sufficiently large. The effect becomes more pronounced with increasing strength or equivalently, in these cases, with decreasing grain size. The important implication is that it would be difficult to discern a scaling law in grain size when involving samples varying not only in grain size but also in their initial texture. Otherwise, it could be wrongly concluded that Hall-Petch scaling does not occur or that an inverse Hall-Petch, a reduction in strength with smaller grain sizes, does occur.

In summary, we explore the effect of initial texture on scaling of strength with nanograin size in nanocrystalline (NC) Cu and Ni using a crystal plasicity based mechanics model. The only plastic deformation mechanism within the model occurs via the statistical emission of dislocations from grain boundaries. The distribution of source lengths in the grain boundaries and the distance that dislocations travel in the grain are both limited by grain size. The model predicts a Hall-Petch strength scaling for all textures studied, from a non-textured material (random) to those typical of electro-deposited NC materials and those made by severe plastic deformation. Good agreement is achieved with numerous reported data sets in the literature. We show that the Hall-Petch slope depends sensitively on texture and is proportional to the Taylor factor. An important consequence is that plastic anisotropy in yield and flow stresses, resulting from texture, will be enhanced as the nanocrystalline grain size decreases. These findings can provide valuable insight into improving nanomaterial processing techniques via texture control, a highly influential, previously overlooked aspect of the nanostructure.

## Methods

In this study, a discrete CPFE model is implemented into finite element software package Abaqus CAE in conjunction with a user-defined material subroutine (UMAT) originally developed by Marin etc.^34^. The model consists of 20 × 20 × 20 grains, wherein each grain represented by an eight-node brick finite element with reduced integration (C3D8R). The kinematics of crystal deformation is based on the multiplicative decomposition of the deformation gradient **F**:


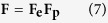


where 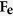 is elastic deformation gradient and 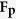 is plastic deformation gradient. The plastic deformation evolves as


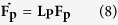


The key modification made here concerns the plastic part of the velocity gradient,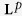. It accounts for the plastic deformation generated by dislocation glide on crystallographic slip planes and is taken to be the summation of the plastic flow over all slip systems as


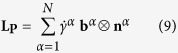


where **b**^*α*^ and **n**^*α*^ are the slip direction and slip plane normal on slip system *α*, 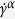 is the shear strain rate on slip system *α*, and *N* is the number of slip systems. The form of 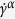 is adapted for statistical emission of dislocations from grain boundaries and is described in Section of Discrete-CPFE.

## Additional Information

**How to cite this article**: Yuan, R. *et al*. Coupled crystal orientation-size effects on the strength of nano crystals. *Sci. Rep.* **6**, 26254; doi: 10.1038/srep26254 (2016).

## Figures and Tables

**Table 1:** Values of *m*_T_ for different textures.

**Texture**	**Random**	**Deposited (100)**	**Deposited (110)**	**Deposited (111)**	**SPD (112)**
x-compression	2.85	2.66	2.93	2.99	3.55
z-compression	2.85	2.42	2.97	3.44	3.01
